# Is Moss Still a Reliable Biomonitor of Nitrogen and Sulfur Deposition After Decades of Emissions Reductions?

**DOI:** 10.3390/plants14071114

**Published:** 2025-04-03

**Authors:** Mehriban Jafarova, Julian Aherne, Monia Renzi, Serena Anselmi, Inga Zinicovscaia, Nikita Yushin, Ilaria Bonini, Stefano Loppi

**Affiliations:** 1 Department of Life Sciences, University of Siena, 53100 Siena, Italy; ilaria.bonini@unisi.it (I.B.); stefano.loppi@unisi.it (S.L.); 2 School of Environment, Trent University, Peterborough, ON K9L 0G2, Canada; 3 Department of Life Sciences, University of Trieste, 34127 Trieste, Italy; mrenzi@units.it; 4 Bioscience Research Center, 58015 Orbetello, Italy; serena.anselmi@bsrc.it; 5 Joint Institute for Nuclear Research, 141980 Dubna, Russia; inga@jinr.ru (I.Z.); ynik_62@mail.ru (N.Y.); 6 National Biodiversity Future Center, 90133 Palermo, Italy

**Keywords:** wet deposition, dry deposition, EMEP, *Hypnum cupressiforme*, Tuscany, Central Italy

## Abstract

Mosses are widely used as biomonitors of atmospheric nitrogen (N) and sulfur (S) deposition due to their broad distribution, ease of sampling, and capacity to trap and accumulate atmospheric particles. However, since 2000, S emissions have decreased by more than 80% across Europe, and N oxides by 40–50%. This study evaluated whether moss remains effective for monitoring atmospheric N and S deposition after decades of emission reductions. This assessment was conducted at 33 rural sites in Tuscany, Central Italy, a region characterized by relatively low levels of N and S deposition. The content of N and S in moss were compared with the air concentrations (gases and particles) and wet and dry deposition of N and S from the Cooperative Programme for Monitoring and Evaluation of Long-range Transmission of Air Pollutants in Europe (EMEP) model and an air pollution index derived from trace element concentrations. The average N content of moss (1.15 ± 0.42%) was an order of magnitude greater than that of S (0.11 ± 0.02%), reflecting the dominance of N deposition. Nevertheless, N and S in moss were strongly correlated (r_s_ = 0.55), suggesting shared sources. Further, N showed a strong correlation with the dry deposition of oxidized N (r_s_ = 0.53), while S was strongly correlated with the wet deposition of S oxides (r_s_ = 0.53) and magnetic susceptibility (r_s_ = 0.69). Overall, our findings confirm that mosses remain effective biomonitors of N and S deposition and can provide reliable spatial and temporal monitoring, especially as the traditional monitoring networks decline.

## 1. Introduction

It is well established that anthropogenic emissions of atmospheric sulfur and nitrogen during the last decades of the twentieth century resulted in the widespread acidification and eutrophication of ecosystems [1]. Accordingly, international political consensus during the 1980s led to the development of emission control policies, e.g., the Gothenburg Protocol under the UNECE Convention on Long-Range Transboundary Air Pollution [1]. Since 2000, emissions of sulfur dioxide have declined by more than 80% across Europe. Similarly, emissions of nitrogen oxides declined by 48%, and ammonia declined by 12% between 2000 and 2020 [2,3,4]. As a consequence, the deposition of sulfur and nitrogen has declined across Europe, leading to ecosystem recovery [2,5,6]. Nonetheless, the continued monitoring of air and precipitation is fundamental to assessing ongoing impacts on ecosystems [7]. However, current networks are spatially challenged, globally and across Europe, in maintaining and developing monitoring activities [7,8]. While atmospheric chemical transport models, such as the Cooperative Programme for Monitoring and Evaluation of Long-range Transmission of Air Pollutants in Europe (EMEP) model [9], have successfully addressed some of these spatial gaps, more precise estimates of deposition are needed to evaluate the effects on ecosystem structure and function, especially on a local scale [10].

Mosses, lacking true roots, derive their nutrients directly from wet and dry atmospheric deposition. As a result, mosses have the capacity to trap and accumulate atmospheric pollutants [11,12]. They have been widely used as biomonitors for assessing the atmospheric deposition of nitrogen and sulfur [13,14,15,16], trace elements [17,18,19], and recently also microplastics [20,21,22]. Further, in areas without naturally growing moss, transplants or moss bags have been successfully used as an effective alternative monitoring tool [23,24]. Moss biomonitoring offers several advantages over the conventional methods, including the ability to simultaneously monitor multiple contaminants using the same sample, as well as simplicity, reliability, and convivence as there is no need for electricity. Additionally, moss biomonitoring is a cost-effective approach that supports the establishment of spatially dense sampling networks, providing the comprehensive preliminary assessment of air quality along scales. However, during the last four decades, there have been significant reductions in pollutant emissions across Europe; as such, it is unknown whether mosses continue to be reliable biomonitors of nitrogen and sulfur deposition.

The objective of this study was to assess whether mosses are still effective biomonitors of atmospheric nitrogen (N) and sulfur (S) deposition following four decades of emission reductions. Our assessment was carried out at 33 rural sites across Tuscany, Central Italy, during April–June 2021. Here, we use modelled air concentration and deposition data from the EMEP model during 2019 and 2020 to estimate site-specific atmospheric N and S. The same study sites were also used in pollution source identification assessment [25], which found that atmospheric cadmium and zinc from long-range atmospheric transport were the main pollutants at these sites.

## 2. Results

**Figure 1:**
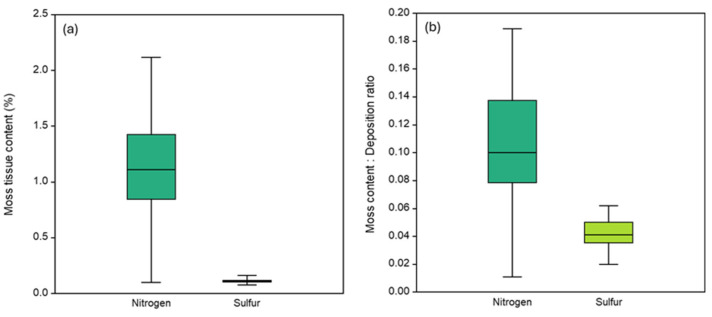
(**a**) The contents (%) of nitrogen and sulfur in the moss, and (**b**) the ratio of moss content (mg N or S/m^2^) and total (wet and dry) atmospheric deposition (mg/m^2^) across the 33 study sites, Tuscany, Central Italy.

The average accumulation of N in the moss samples (1.15 ± 0.42%) was more than ten times higher than that of S (0.11 ± 0.02%) across the study sites (Table 1 and Figure 1). Further, the N content was more (ca. twofold) spatially variable (36.5%) compared with S (17.4%; Table 1). Nonetheless, the N and S contents were significantly correlated (r_s_ = 0.54, *p* < 0.05), suggesting that they have a similar source, or that the moss takes up N and S at a fixed ratio of 10:1. On an area basis, the average moss content was 8.4 mg/m^2^ for S and 84.9 mg/m^2^ for N (Table 1).

The average total (wet and dry) deposition of N (822 ± 151 mg/m^2^) was almost four times higher than total S deposition (214 ± 66 mg/m^2^) across the study sites during 2019–2020 (Table 1). Further, total deposition was dominated by wet deposition for both N (66%) and S (86%), and N deposition was almost equally dominated by reduced (51%) and oxidized (49%) N (see Table S1). In contrast to the moss S content, total S deposition was more spatially variable (31%) compared with total N deposition (18.3%; Table S1). However, the spatial variation in total oxidized N deposition (28.5%; Table 1) was similar to that of total S deposition. The deposition variables were strongly correlated (Figure S1); total S deposition was correlated to the wet deposition of oxidized S (r_s_ = 0.96, *p* < 0.05) and the total deposition of oxidized S (r_s_ = 0.93, *p* < 0.05). Further, total N deposition was correlated to the wet deposition of N (r_s_ = 0.85, *p* < 0.05) and the total deposition of reduced N (r_s_ = 0.82, *p* < 0.05). The ratio of moss content (mg/m^2^) to total deposition was 0.10 for N and 0.04 for S (Table 1 and Figure 1).

**Figure 2:**
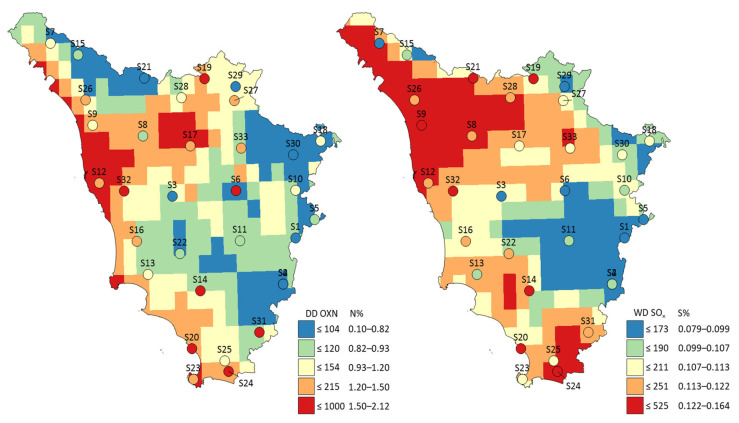
(**Left**) Dry deposition of oxidized nitrogen (DD OXN; 2019–2020) overlayed with nitrogen content (N%; filled circles) in moss at study sites (*n* = 33), and (**right**) wet deposition of sulfate (WD SO_×_; 2019–2020) overlayed with sulfur content (S%; filled circles) in moss at study sites across Tuscany, Central Italy.

The contents of N and S in the moss were significantly correlated to atmospheric N and S (Figures S2 and S3). The nitrogen content in the moss was significantly correlated with the dry deposition of oxidized N (r_s_ = 0.53, *p* < 0.05) and the air concentration of coarse particulate nitrate (r_s_ = 0.53, *p* < 0.05). Further, the sulfur content in the moss was significantly correlated with the wet deposition of oxidized S (r_s_ = 0.53, *p* < 0.05) and the total deposition of S (r_s_ = 0.53, *p* < 0.05), which was dominated by the wet deposition of oxidized sulfur (86%; Table S1). Surprisingly, the N and S contents in the moss were both correlated with the oxidized forms of N and S deposition, further suggesting similar sources. In general, there was a spatial coherence between atmospheric deposition and the moss content (Figure 2 and Figure S4). Perhaps more surprisingly, the N content in the moss was not correlated to reduced N deposition, suggesting that the oxidized forms of N are preferentially taken up by moss or that the EMEP model estimates of reduced N deposition are less accurate.

**Figure 3:**
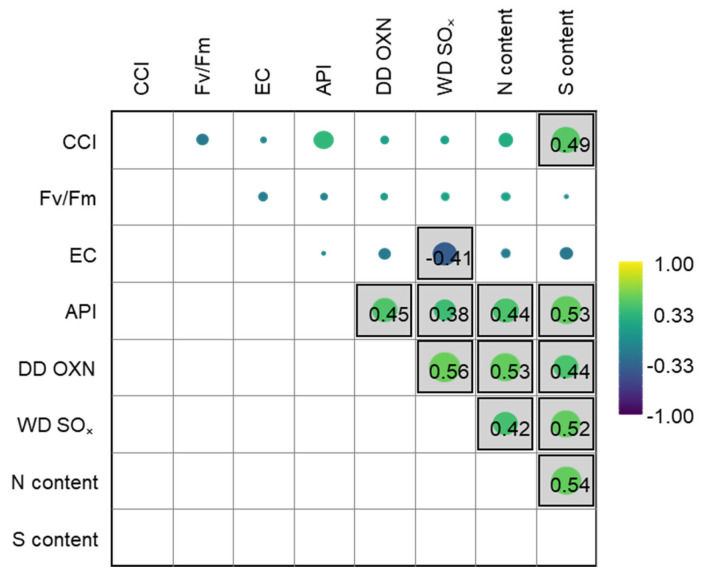
Spearman’s rank correlation matrix for nitrogen (N) and sulfur (S) contents (%) in moss against wet deposition of oxidized sulfur (WD SO_x_), dry deposition of oxidized nitrogen (DD OXN), air pollution index (API), and moss vitality (total chlorophyll content (CCI), photosynthetic efficiency (F_v_/F_m_), and cell membrane integrity (EC)). Boxes indicate significant (*p* < 0.05) correlation.

The contents of N and S in the moss were significantly correlated with anthropogenic air pollution across the study sites [22], as expressed by the API (r_s_ = 0.44 and r_s_ = 0.53, *p* < 0.05, for N and S, respectively; Figure 3). This further suggests that the source of N and S in the moss was associated with atmospheric deposition, predominantly with oxidized N and S. Further, redundancy analysis suggested that the other environmental variables (population, land cover, distance to road, etc.) had a limited influence on the N and S contents in the moss (see Figure S5). Surprisingly, the S content was correlated with the CCI (r_s_ = 0.49, *p* < 0.05), suggesting that atmospheric deposition may not have a negative impact on moss vitality.

## 3. Discussion

In general, the contents of N and S in the moss in our study (1.15% and 0.11%, respectively) were comparable with those in previously published studies. The moss content of N across 14 countries in Europe ranged from 1.28% in 2005 to 1.21% in 2010, with the ~5% decline between 2005 and 2010 associated with emission reductions [14]. Similarly, in Italy (Bolzano), the moss (*Hypnum cupressiforme*) content of N across 20 sites ranged from 1.12% in 2005 to 1.09% in 2010 [14]. Further, moss (*H. cupressiforme*) collected from a rural area in northeastern Italy near Basovizza, Trieste, in 2003 had a content of N (1.26%) and S of 0.10% [26]. The mean S content in moss (*Pleurozium schreberi*) across National Parks in Poland ranged from 0.09–0.21% in 1975 to 0.11–0.24% in 1986 [27]. During the early 1990s, the S content in moss (*Sphagnum fuscum*) across 28 sites in Canada was 0.12 ± 0.06% [28], and during the early 2000s, the N and S contents in moss (*Isothecium myosuroides*) across 45 sites in southwestern British Columbia were 1.35 ± 0.40% and 0.10 ± 0.02%, respectively [29]. In general, the slightly higher content observed in the European studies in the last decade highlight a significant decline in N and S deposition across Europe [2], and also in Italy [30] due to strict emission control measures during the last two decades. However, atmospheric deposition also varies greatly between regions and continents, which explains the differences in moss content. Nonetheless, despite emission controls, current N deposition across the study area was elevated. The average background concentration of N in mosses is estimated to be 0.5–0.6% [31,32]; in our study, only one site was within this threshold, suggesting that this region experiences elevated N deposition. Further, it has been suggested that an S:N ratio <0.15 indicates N excess in moss [33]; in our study, the average ratio was 0.13, and only four sites had ratios above this threshold.

Numerous studies have noted strong (significant) associations between the moss content of N and bulk (or total) N deposition [13,14,32,34,35,36]. In the current study, atmospheric deposition and the indicators of air pollution (i.e., API) had the strongest associations with the moss contents of N and S. A weak correlation with environmental variables (see Figure S5) has previously been observed across Europe, i.e., weak correlations (r_s_ < 0.5) were observed for altitude, precipitation, the distance to sea, forested land use, and population density [36]. In the same study, oxidized N in dry (r_s_ = 0.64) and wet deposition (r_s_ = 0.65) had a strong correlation with the N content in the moss, but the air concentrations of ammonium showed a stronger correlation (r_s_ = 0.68). Similarly, in Scotland, the N content in the moss was an indicator of N deposition at sites, where deposition was dominated by ammonia [37], although there is a stronger relationship with the nitrate concentrations in rain (R^2^ = 0.69) compared with those of ammonium (R^2^ = 0.57). In contrast, in our study, the moss was correlated only with oxidized N deposition, not reduced N, and this may indicate that the recent higher spatial resolution modelling of reduced N in the EMEP model [9] may not accurately reflect deposition across Tuscany. It is well established that there is good agreement between the EMEP and observed sulfate and nitrate deposition; however, larger differences have been observed for ammonium (reduced) deposition [10]. Alternatively, this suggests that moss preferentially takes up oxidized N over reduced N deposition, which may be an adaptive response to avoid N intoxication [38].

The current atmospheric deposition of N and S across Tuscany did not appear to affect the vitality of mosses. During the last two decades, there have been large reductions in the emission and deposition of N and S across Europe and Italy [2,30]. As such, S deposition did not negatively affect the chlorophyll content of the moss; rather, it appeared to support it, as evidenced by the positive correlation between the S content and the CCI (r_s_ = 0.49). Similarly, several studies have demonstrated that S promotes the chlorophyll content in various plants, while its deficiency can lead to a reduction in chlorophyll levels [39]. Although some studies suggest that S exposure can decrease the chlorophyll content in moss [40], our findings suggest that low S deposition has a positive effect on chlorophyll levels.

Our results demonstrate that moss continues to be an effective biomonitor of N and S deposition despite four decades of emission reductions across Europe. Moreover, the moss showed sensitivity to the variations in atmospheric N and S levels, offering valuable insights into the spatial distribution of these pollutants. Further, this suggests that moss can effectively reflect changes in pollution trends over time, which is particularly useful in areas that are challenging to monitor using the conventional methods. Our findings highlight the enduring significance of moss as an affordable and dependable tool for tracking atmospheric N and S deposition.

## 4. Materials and Methods

### 4.1. Study Area and Sampling Sites

**Figure 4:**
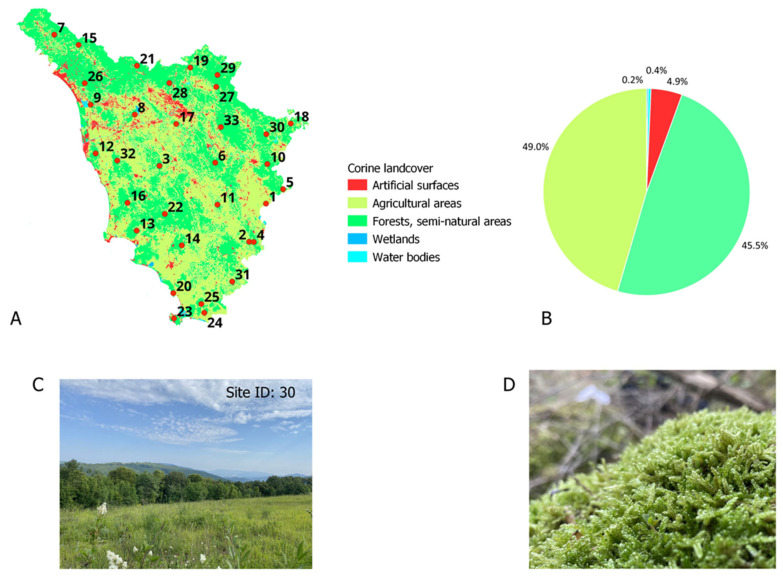
Map showing study sites and land cover of Tuscany, Central Italy ((**A**) 33 moss sampling sites across study area underlain by Corine land cover classes; (**B**) pie chart showing land cover proportion with 49.0% agricultural areas, 45.5% forests, and semi-natural areas, 4.9% artificial surfaces, 0.4% water bodies, and 0.2% wetlands; (**C**) image of sampling site 30; (**D**) image of dominant moss species, *Hypnum cuppressiforme*).

Moss samples were collected from 33 rural sites across Tuscany, Central Italy (Figure 4). Site selection followed the moss survey protocol established by the International Cooperative Programme (ICP) on the impacts of air pollutants on crops and (semi-)natural vegetation [41]. Using QGIS and the Corine Land Cover 2000 classification, the sites were randomly chosen based on specific criteria: a minimum distance of 300 m from major roads, villages, and industrial areas (including farming) and at least 100 m from minor roads and houses. Further, all the sites were located far from direct sources of air pollution. In general, the sites were randomly distributed within a 50 km × 50 km EMEP grid [14], with an average of two sampling sites per grid cell (Figure S6). Tuscany’s land cover consists mainly of agricultural areas (49%) and forests (46%); in this study, all the sampling sites were open stands situated in forested and semi-natural areas (Figure 4) at an elevation in the range 46–1079 m asl (Table S2). Tuscany has a sub-Mediterranean climate characterized by mild winters; warm, dry summers; an average annual temperature of 13–14 °C; and annual rainfall in the range 600–1000 mm.

### 4.2. Moss Sampling

Since it proved impossible to find the same pleurocarpous moss at each site, overall, eight species were collected during April–June 2021, namely *Hypnum cupressiforme*, *Pseudoscleropodium purum*, *Homalothecium sericeum*, *Eurynchium striatulum*, *Eurynchium striatum*, *Brachitecium rutabulum*, *Isothecium alopecuroides*, and *Hylocomium splendens* (Figure S7), with *H. cupressiforme* being by far the most abundant (64% of sites). At each site, five samples (replicates) were collected from a 50 m × 50 m plot, with a distance of at least 5 m to the nearest projected tree canopy. The samples were put into paper bags, closed tightly, and stored in a freezer until analysis. A total of 165 moss samples (33 sites × 5 replicates) were analyzed for total N and S concentration (%) and vitality parameters (total chlorophyll content, photosynthetic efficiency, and cell membrane integrity). Assessing possible interspecies differences was out of the scope of this study.

### 4.3. Nitrogen and Sulfur Analysis

The moss samples were cleaned of debris and carefully washed for a few seconds with 100 mL of deionized water to clean possible soil contamination. The washed samples were dried to a constant weight at 105 °C, homogenized using a planetary mill (Pulverisette 6, Fritsch, Idar-Oberstein, Germany) and analyzed for N using a combustion analyzer (LECO Instrument GmbH, Mönchengladbach, Germany) and for S using Inductively coupled plasma-Optical Emission Spectrometry (ICP-OES, PlasmaQuant 9000 Elite, Analytik Jena, Jena, Germany), following microwave acid digestion (Mars 6, CEM, Matthews, NC, USA) according to [42]. Oriental Basma Tobacco Leaf (INCT-OBTL-5) reference material was used for quality control; the recovery of elements ranged from 96% to 110%. Calibration solutions and standards were prepared from ICP multi-element standard solution (Sigma-Aldrich, Darmstadt, Germany) and were analyzed after every 10 samples.

### 4.4. Sample Vitality

To evaluate the vitality of the mosses, their physiological parameters (total chlorophyll content and chlorophyll a fluorescence) and cell membrane integrity were examined. Five measurements were taken for each replicate sample, randomly selecting the moss shoots to measure.

The total chlorophyll content index (CCI), expressed as chlorophyll content per square meter of biological material (mg/m^2^), was evaluated using a chlorophyll content meter (CCM-300, Opti-Science, Hudson, NH, USA).

Photosynthetic efficiency was assessed using the widely used indicator F_v_/F_m_, which estimates the maximum quantum yield of photosystem II (PS II) photochemistry. Prior to analysis, the mosses were dark-adapted for 15 min. Analysis was carried out by flashing the samples with a saturating (3000 µmol/m^2^/s) red light (650 nm) pulse for one second using a plant efficiency analyzer (Handy PEA, Hansatech Ltd., Norfolk, UK).

The integrity of cell membranes in the moss samples was assessed through a step-by-step stress evaluation process [43]. Initially, the moss samples weighing 100 mg were soaked in 50 mL of deionized water and shaken for 1 h, after which the electrical conductivity (EC, S/m) of the resulting solution was measured using a conductivity meter (Basic 30, Crison, Barcelona, Spain). Then, the samples were heat-stressed at 90–100 °C to cause the complete rupture of cell membranes, after which EC was measured again. The results are expressed as % between the first and the second EC readings.

### 4.5. Data Analysis

The total contents of N and S in moss samples are expressed as % and on a surface area basis (mg/m^2^). For the latter, the mass data (mg/g = % × 10) were converted into surface area (mg/m^2^) using a specific leaf area of 0.135 m^2^/g [44,45].

Modelled air concentrations of N and S gases and particles, and the wet and dry deposition of N and S (21 variables; see Figures S2 and S3) across Tuscany, Central Italy, for the 2019–2020 period (two years prior to the 2021 sampling year) were obtained from the EMEP [46] and extracted for each sampling site. The ratio of total moss content (mg N or S/m^2^) to total atmospheric deposition (mg N or S/m^2^) was estimated for each sampling site (n = 33).

The association between atmospheric N and S and their content in moss was assessed using Spearman’s rank correlation coefficient (r_s_; only significant (*p* < 0.05) associations are reported). The atmospheric N and S variables with the strongest correlation were mapped (Surfer 21.2, Golden Software) across the region together with the contents (%) of N and S in moss at the 33 study sites (see Figure 2 and Figure S4).

The source identification of N and S in the moss employed a number of existing data sets. The air pollution index (API) was obtained for the 33 study sites from Jafarova et al. [25]. The API was derived from the factor analysis of potentially toxic element concentrations in the moss samples and represents anthropogenic air pollution from long-range atmospheric transport.

The influence of environmental variables on the N and S contents (%) in the moss were further assessed using redundancy analysis (RDA), which evaluates the relationship between the response (N and S content) and the explanatory variables (environmental predictors: population density in 5 and 10 km buffers, land cover, altitude, building count in 500 m buffer, distance to main roads, moss vitality, API, and atmospheric N and S data). A 500 m buffer was established around each sampling site, within which buildings were tallied, and the dominant landscape was recorded (village or town, agricultural, pasture, heathland, scrub woodland, or forest) using Google Earth Pro. Subsequently, the distance (m) from the sampling site to the nearest road was measured, and population densities within both 5 km and 10 km buffers were further estimated (public.opendatasoft.com). All statistical analyses was performed using the free software R 4.4.3 [47] and Past 4.15 [48].

## Figures and Tables

**Table 1:** Mean ± standard deviation, median, minimum (min), and maximum (max), and coefficient of variation (CV) for contents of nitrogen (N) and sulfur (S) accumulated in moss samples (% and mg/m^2^); total (wet and dry) atmospheric deposition of N and S, and oxidized nitrogen (OXN) deposition (mg/m^2^) during 2019–2020; moss-content-to-deposition ratios; and moss vitality (CCI—chlorophyll content index (mg/m^2^); F_v_/F_m_—photosynthetic efficiency; EC—electric conductivity ratio) across 33 rural sites, Tuscany, Central Italy.

Variable	Mean ± Std	Median	Min.	Max.	CV (%)
N content (%)	1.15 ± 0.42	1.11	0.10	2.12	36.5
S content (%)	0.11 ± 0.02	0.11	0.08	0.16	17.4
N content (mg/m^2^)	84.9 ± 31.0	82.2	7.7	157.0	36.5
S content (mg/m^2^)	8.4 ± 1.5	8.2	5.8	12.2	17.4
N Deposition (mg/m^2^)	822 ± 151	789	651	1205	18.3
OXN Deposition (mg/m^2^)	406 ± 116	380	270	699	28.5
S Deposition (mg/m^2^)	214 ± 66	203	142	423	31.0
N content: Deposition	0.10 ± 0.04	0.10	0.01	0.19	38.3
S content: Deposition	0.04 ± 0.01	0.04	0.02	0.06	24.6
CCI (mg/m^2^)	438 ± 42	444	325	523	9.6
F_v_/F_m_	0.66 ± 0.03	0.66	0.58	0.74	4.8
EC (%)	10.5 ± 3.79	9.47	5.37	25.3	36.0

## Data Availability

The original contributions presented in this study are included in the Supplementary Materials.

## Supplementary Materials

The following supporting information can be downloaded at: https://www.mdpi.com/article/10.3390/plants14071114/s1, Figure S1: Association (Spearman’s rank correlation) between nitrogen (N) and sulfur deposition, wet deposition (WD), dry deposition (DD), total deposition (TD), and reduced (RDN) and oxidized (ONX) nitrogen across the 33 study sites in Tuscany, Central Italy. Boxes indicate significant (*p* < 0.05) correlation. Figure S2: Association (Spearman’s rank correlation) between air concentration and deposition of nitrogen (N) across the 33 study sites in Tuscany, Central Italy. Boxes indicate significant (*p* < 0.05) correlation. Figure S3: Association (Spearman’s rank correlation) between air concentration and deposition of sulfur (S) across the 33 study sites in Tuscany, Central Italy. Boxes indicate significant (*p* < 0.05) correlation. Figure S4. Spatial distribution of (left) nitrogen content (N) in moss, and (right) sulfur content (S) in moss at study sites (n = 33) across Tuscany, Central Italy. Figure S5: Redundancy analysis biplot for moss contents of nitrogen (N) and sulfur (S), with 13 environmental predictors (population in 5 km and 10 km buffers, building number, land cover, distance to main roads (m), altitude (m), moss vitality (total chlorophyll content (CCI), photosynthetic efficiency (F_v_/F_m_), and cell membrane integrity (EC)), air pollution index (API), wet deposition of sulfur (WD SO_x_), and dry deposition of oxidized nitrogen (DD OXN)) as explanatory variables (R^2^ = 0.61; R^2^ adj = 0.34). Figure S6: A map showing the location of the moss sampling sites within the EMEP 50 km × 50 km grid across Tuscany, Central Italy. Figure S7: Pie chart showing moss species proportions across sites (A—*Hypnum cuppressiforme* (64%), *Pseudoscleropodium purum* (13%), *Brachythecium rutabulum* (8%), *Eurhynchium striatulum* (7%), *Homalethecium sericeum* (4%), *Eurhynchium striatulum* (3%), *Isothecium alopecuroides* (1%), and *Hylocomium splendens* (1%)) collected from 33 sites in Tuscany, Central Italy, and images of the two most dominant moss species (B—*H. cupressiforme*; C—*P. purum*). Table S1: Nitrogen (N) and sulfur (S) deposition in species at 33 study sites in Tuscany, Central Italy. Table S2: The latitude, longitude, altitude, and nitrogen (N) and sulfur (S) contents (%) in the moss and the air pollution index (API) at the 33 rural study sites in Tuscany, Central Italy. Table S3. Association (Spearman’s rank correlation) between air concentrations (SURF) and deposition of nitrogen (N) across the 33 study sites in Tuscany, central Italy.
