# Supplementary material for: Is Moss Still a Reliable Biomonitor of Nitrogen and Sulfur Deposition After Decades of Emissions Reductions?
Source: Plants (Basel). 2025 Apr 3;14(7):1114. doi: 10.3390/plants14071114 (PMC11991018; doi:10.3390/plants14071114)
Supplement: Supplementary file 1 [file plants-14-01114-s001.zip › plants-3538697-supplementary.pdf]

Supplementary Material

Is moss still a reliable biomonitor of nitrogen and sulfur deposition after decades of emissions reductions?

Mehriban Jafarova [1,2]*, Julian Aherne [2], Monia Renzi [3], Serena Anselmi [4], Inga Zinicovscaia [5], Nikita Yushin [5], Ilaria Bonini [1] and Stefano Loppi [1,6]

[1] Department of Life Sciences, University of Siena, 53100 Siena, Italy

[2] School of Environment, Trent University, Peterborough, ON, Canada K9L 0G2

[3] Department of Life Sciences, University of Trieste, Italy

[4] Bioscience Research Center, Orbetello, Italy

[5] Joint Institute for Nuclear Research, Dubna, Russia

[6] National Biodiversity Future Center, Palermo, Italy

* Corresponding author: Mehriban Jafarova

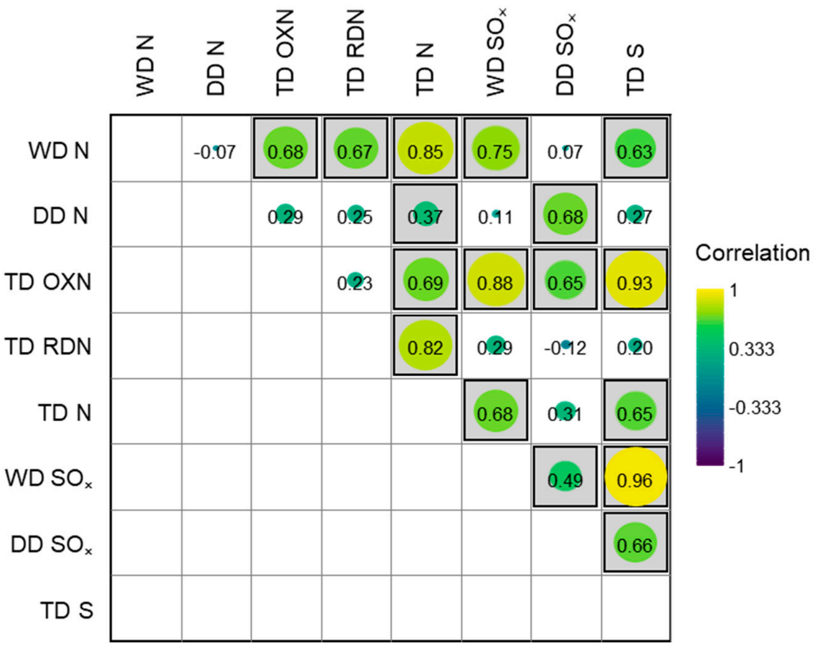

Figure S1. Association (Spearman's rank correlation) between nitrogen (N) and sulfur (S) deposition, wet deposition (WD), dry deposition (DD), total deposition (TD), and reduced (RDN) and oxidized (ONX) nitrogen across the 33 study sites in Tuscany, central Italy. Boxes indicate significant ($p < 0.05$) correlation.

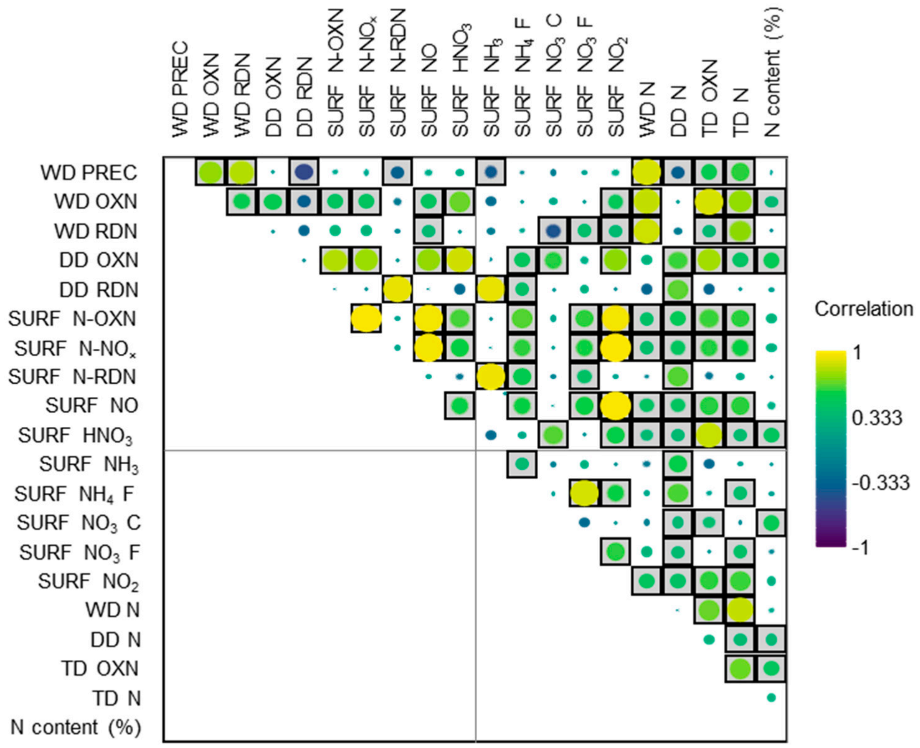

Figure S2. Association (Spearman's rank correlation) between air concentrations (SURF) and deposition of nitrogen (N) across the 33 study sites in Tuscany, central Italy. Boxes indicate significant ($p < 0.05$) correlation. Variables are described below. See Table S3 for the correlation coefficients.

WD PREC	Precipitation volume
WD OXN	Wet deposition of oxidized nitrogen
WD RDN	Wet deposition of reduced nitrogen
DD OXN	Dry deposition of oxidized nitrogen
DD RDN	Dry deposition of reduced nitrogen
SURF N-OXN	Air concentration of total oxidized nitrogen
SURF N-NO×	Air concentration of nitrogen oxides
SURF N-RDN	Air concentration of total reduced nitrogen
SURF NO	Air concentration of nitric oxide
SURF HNO3	Air concentration of nitric acid
SURF NH3	Air concentration of ammonia
SURF NH4 F	Air concentration of fine particulate ammonium
SURF NO3 C	Air concentration of coarse particulate nitrate
SURF NO3 F	Air concentration of fine particulate nitrate
SURF NO2	Air concentration of nitrogen dioxide
WD N	Wet deposition of nitrogen
DD N	Dry deposition of nitrogen
TD OXN	Total deposition of oxidized nitrogen
TD N	Total deposition of nitrogen
N content	Nitrogen content in moss

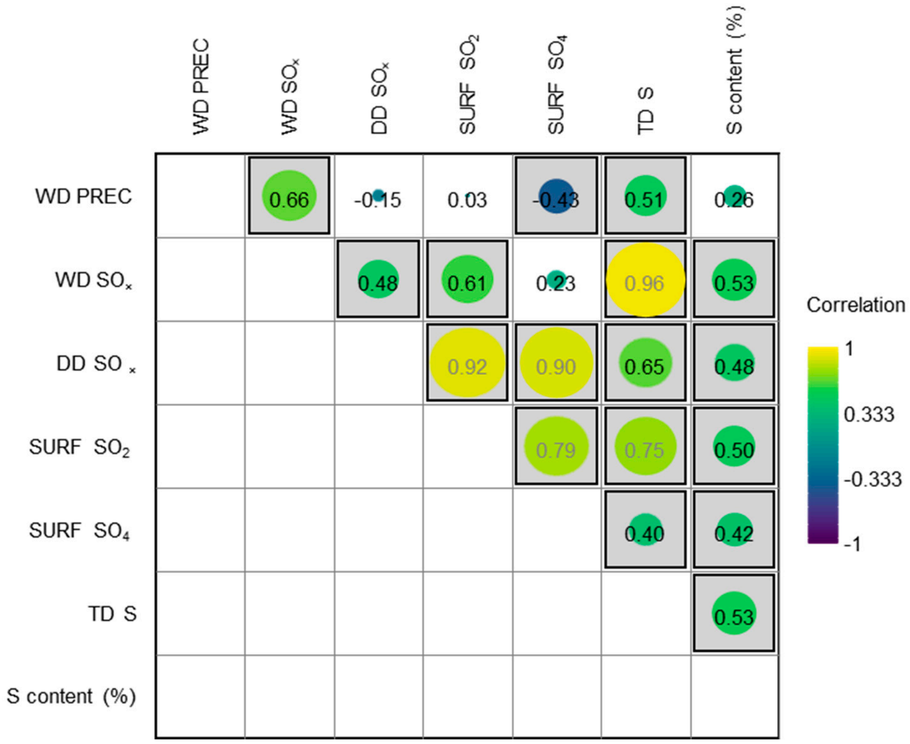

Figure S3. Association (Spearman's rank correlation) between air concentrations and deposition of sulfur (S) across the 33 study sites in Tuscany, central Italy. Boxes indicate significant ($p < 0.05$) correlation. Variables are described below.

WD PREC	Precipitation volume
WD SO_x	Wet deposition of oxidized sulfur
DD SO_x	Wet deposition of sulfate
SURF SO_2	Air concentration of sulfur dioxide
SURF SO_4	Air concentration of particulate sulfate
TD S	Total deposition of sulfur
S content	Sulfur content in moss

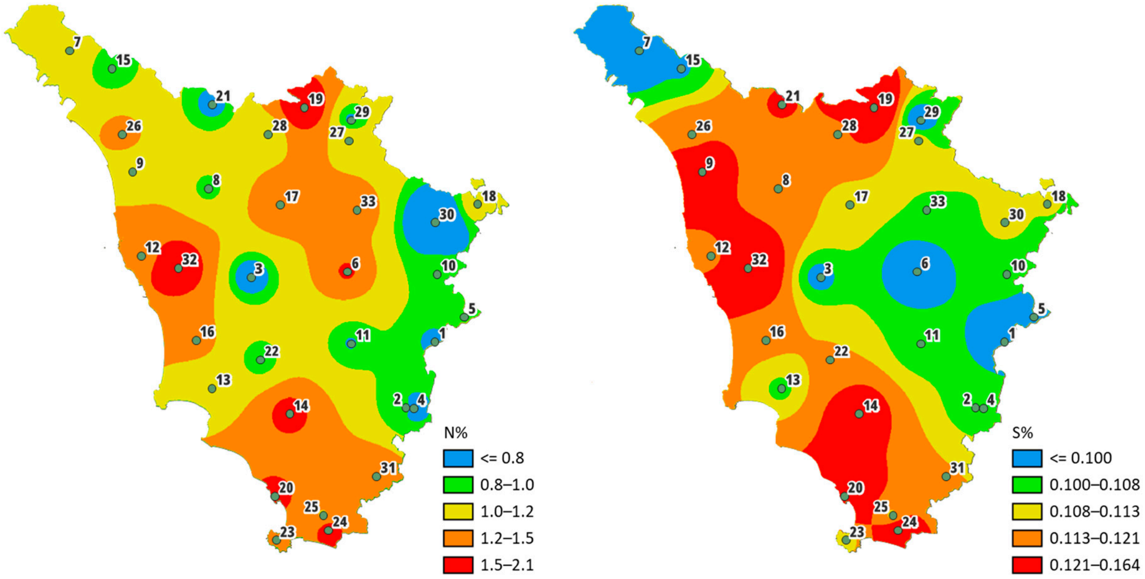

Figure S4. Spatial distribution of (left) nitrogen content (N) in moss, and (right) sulfur content (S) in moss at the study sites (n = 33) across Tuscany, Central Italy.

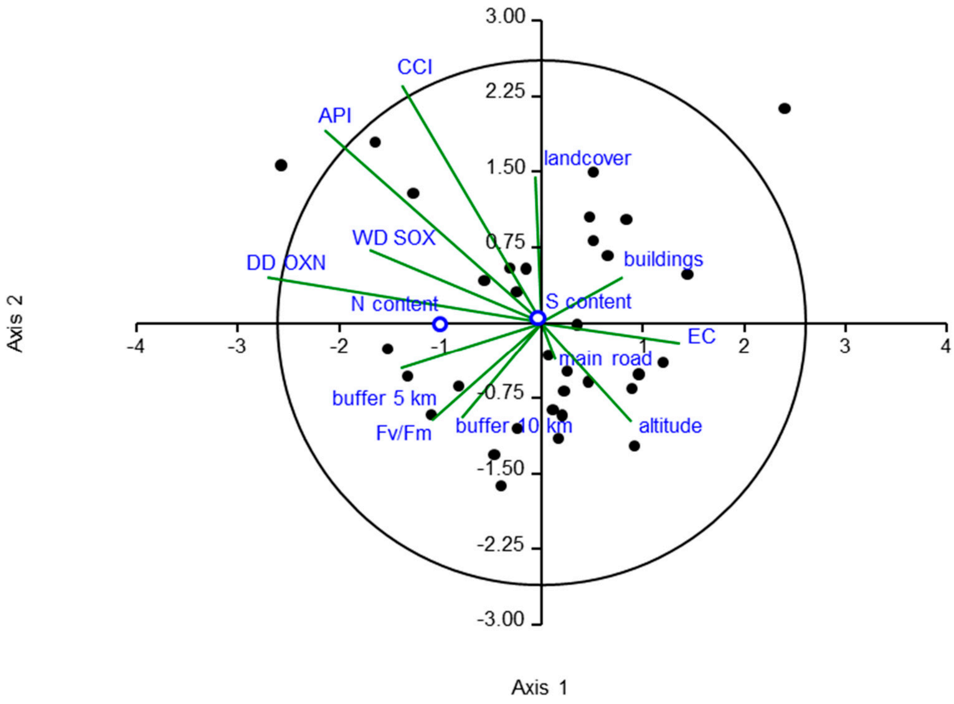

Figure S5. Redundancy Analysis biplot for moss content of nitrogen (N) and sulfur (S), with the 12 environmental predictors (population in 5 km and 10 km buffers, building number, land cover, distance to main roads (m), altitude (m), moss vitality (total chlorophyll content (CCI), photosynthetic efficiency (Fv/Fm), and cell membrane integrity (EC)), air pollution index (API), wet deposition of sulfur (WD SO_x), and dry deposition of oxidized nitrogen (DD OXN)) as explanatory variables (R^2 = 0.61 ; R^2 adj = 0.34).

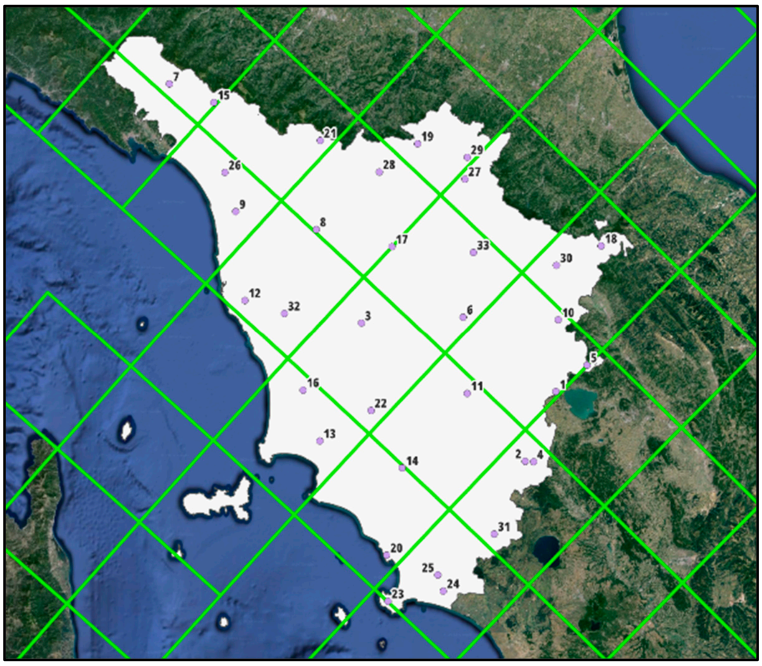

Figure S6. Map showing the location of the moss sampling sites within the EMEP 50 km × 50 km grid across Tuscany, central Italy.

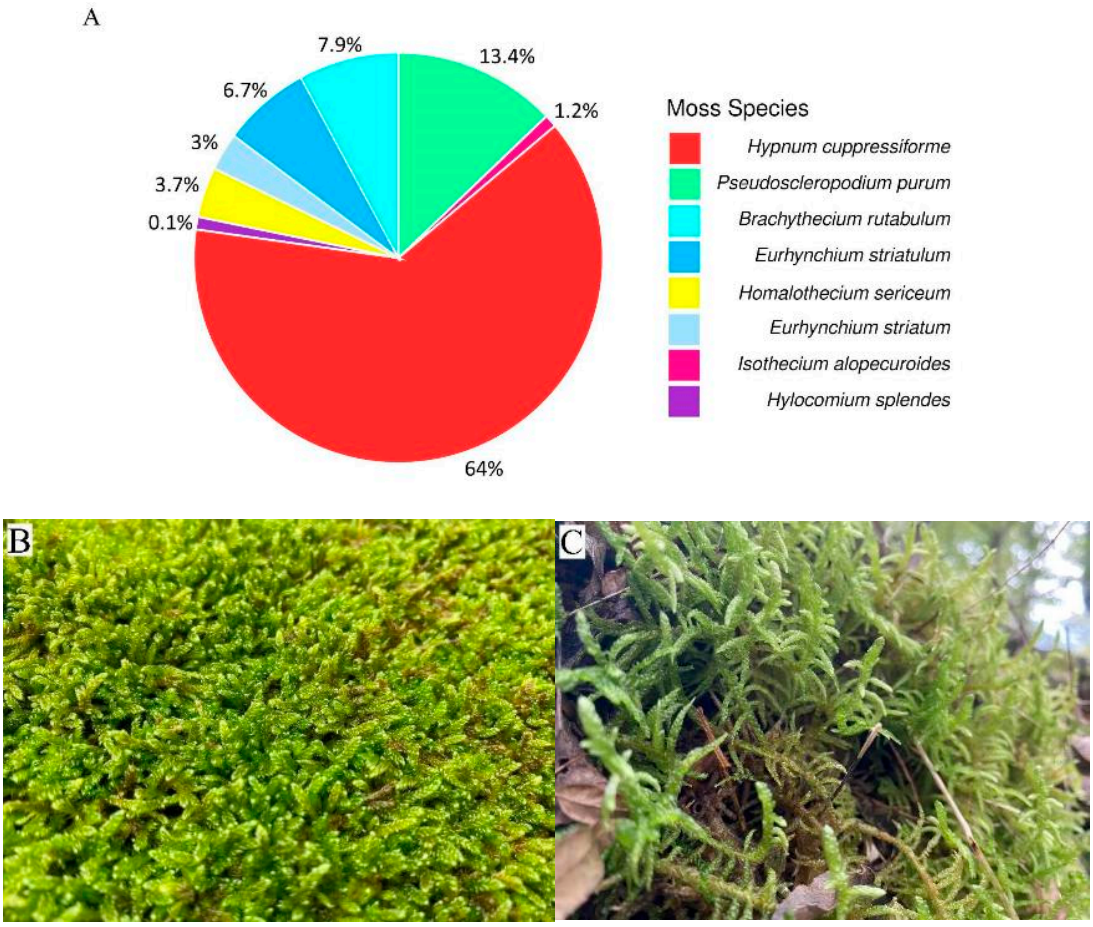

Figure S7. Pie chart showing moss species proportions (A – *Hypnum cuppressiforme* (64%), *Pseudoscleropodium purum* (13%), *Brachythecium rutabulum* (8%), *Eurhynchium striatulum* (7%), *Homalethecium sericeum* (4%), *Eurhynchium striatulum* (3%), *Isothecium alop~~p~~ecuroides* (1%), and *Hylocomium splendens* (1%)) collected from 33 sites in Tuscany, central Italy, and images of the two most dominant moss species (B – *H. cuppressiforme*, and C – *P. purum*).

Table S1. Nitrogen (N) and sulfur (S) deposition species at the 33 study sites in Tuscany, central Italy. See Figure S2 and S3 for a description of the abbreviations; further, %WD N is the percentage of total N deposition that is wet deposition; %WD S is the percentage of total S deposition that is wet deposition.

ID	WD N mg/m^2	TD OXN mg/m^2	TD RDN mg/m^2	TD N mg/m^2	%WD N %	WD SO_x mg/m^2	TD S mg/m^2	%WD S %
1	457.8	283.6	646.6	930.3	49.2	122.1	145.6	83.8
2	398.6	269.9	382.6	652.6	61.1	123.2	144.4	85.3
3	432.1	336.4	355.6	692.0	62.5	149.0	176.8	84.3
4	398.6	270.0	382.8	652.8	61.1	123.2	144.4	85.3
5	502.0	308.9	519.4	828.3	60.6	139.9	161.5	86.7
6	447.0	315.3	366.5	681.8	65.6	143.4	163.8	87.6
7	827.8	582.6	486.5	1069.1	77.4	257.3	279.0	92.2
8	698.9	574.3	477.7	1052.0	66.4	228.9	271.4	84.3
9	892.6	699.0	505.7	1204.6	74.1	319.5	364.8	87.6
10	476.1	313.1	417.8	730.9	65.1	149.7	169.6	88.3
11	372.7	275.4	375.9	651.3	57.2	120.7	142.2	84.9
12	582.7	631.8	395.5	1027.4	56.7	264.9	422.7	62.7
13	489.4	391.0	338.9	729.8	67.1	178.7	210.4	85.0
14	476.3	366.8	395.1	761.9	62.5	178.2	204.4	87.2
15	611.3	382.2	407.1	789.2	77.4	165.1	179.8	91.8
16	503.5	379.9	376.4	756.3	66.6	165.3	197.4	83.7
17	600.3	533.6	426.5	960.1	62.5	181.1	222.9	81.3
18	540.5	300.6	495.1	795.7	67.9	137.8	156.7	88.0
19	592.0	435.9	461.8	897.7	65.9	164.5	187.7	87.7
20	375.2	395.8	285.5	681.3	55.1	164.0	203.0	80.8
21	646.5	390.7	425.7	816.4	79.2	193.6	207.5	93.3
22	469.3	323.8	349.0	672.8	69.7	160.8	185.3	86.8
23	341.1	432.6	243.5	676.1	50.4	161.6	222.6	72.6
24	510.9	432.1	443.4	875.5	58.4	222.2	262.9	84.5
25	490.2	397.0	383.2	780.2	62.8	206.8	240.8	85.9
26	951.4	658.8	513.2	1172.0	81.2	333.1	358.8	92.8
27	565.8	378.0	444.3	822.3	68.8	164.3	186.9	87.9
28	696.7	500.6	458.9	959.5	72.6	226.2	253.5	89.2
29	531.4	364.1	411.1	775.2	68.6	146.0	168.5	86.6
30	479.2	294.5	387.1	681.6	70.3	145.7	161.5	90.2
31	538.8	374.6	443.9	818.6	65.8	210.1	236.9	88.7
32	514.1	425.2	366.3	791.6	65.0	165.1	209.2	78.9
33	547.2	369.1	382.8	751.9	72.8	184.2	204.4	90.1
Average	544.2	405.7	416.7	822.4	65.7	181.7	213.6	85.6
CV (%)	26.0	28.5	17.9	18.3	11.6	28.9	31.0	6.8

Table S2. The latitude, longitude, altitude, nitrogen (N) and sulfur (S) content (%) in moss, and an Air Pollution Index (API) derived from factor analysis of potentially toxic element concentrations in moss samples (Jafarova et al., 2025) § at the 33 rural study sites in Tuscany, central Italy.

ID	Latitude dms	Longitude dms	Altitude m	N %	S %	API
1	43°10'45.4"N	11°58'33.7"E	200	0.77	0.086	-0.90
2	42°55'29.5"N	11°51'51.1"E	802	0.89	0.104	-0.92
3	43°25'59.5"N	11°00'00.4"E	396	0.62	0.096	-0.86
4	42°55'27.8"N	11°51'53.8"E	492	0.71	0.107	-1.11
5	43°16'39.7"N	12°08'00.1"E	426	0.92	0.091	-0.57
6	43°27'15.3"N	11°30'39.4"E	621	1.55	0.080	-0.33
7	44°17'58.3"N	10°02'06.7"E	660	1.16	0.079	-0.65
8	43°46'23.5"N	10°46'23.4"E	46	0.91	0.113	-0.27
9	43°50'19.5"N	10°22'06.9"E	163	1.07	0.135	-0.56
10	43°26'42.5"N	11°59'19.6"E	780	0.93	0.107	-0.87
11	43°10'27.8"N	11°31'53.5"E	390	0.82	0.102	-0.89
12	43°30'55.9"N	10°24'51.9"E	332	1.39	0.116	0.91
13	43°00'01.3"N	10°47'28.4"E	78	0.99	0.106	-0.67
14	42°54'06.4"N	11°12'17.1"E	278	1.69	0.138	-0.79
15	44°13'56.3"N	10°15'31.5"E	1013	0.84	0.100	0.34
16	43°11'11.4"N	10°42'25.2"E	447	1.28	0.114	1.03
17	43°42'40.6"N	11°09'12.5"E	345	1.44	0.111	0.13
18	43°42'50.2"N	12°12'14.2"E	595	1.11	0.108	-0.65
19	44°05'01.3"N	11°16'56.2"E	894	2.12	0.157	1.84
20	42°34'49.5"N	11°07'36.5"E	128	1.63	0.149	0.98
21	44°05'44.1"N	10°47'37.5"E	1079	0.74	0.122	-0.03
22	43°06'43.4"N	11°02'56.6"E	408	0.85	0.114	0.41
23	42°24'33.1"N	11°07'59.9"E	176	1.28	0.110	1.19
24	42°26'51.4"N	11°24'41.0"E	121	1.64	0.134	1.79
25	42°30'26.0"N	11°23'00.9"E	117	1.17	0.112	-1.03
26	43°58'49.3"N	10°18'53.0"E	467	1.28	0.120	1.37
27	43°57'24.5"N	11°31'07.5"E	290	1.20	0.111	-0.08
28	43°58'54.3"N	11°05'27.0"E	655	1.01	0.119	0.71
29	44°02'05.8"N	11°31'54.8"E	831	0.79	0.091	0.53
30	43°38'35.7"N	11°58'47.3"E	542	0.10	0.109	0.39
31	42°39'37.2"N	11°39'51.0"E	350	1.50	0.113	-0.80
32	43°28'05.4"N	10°36'46.7"E	329	2.02	0.164	0.62
33	43°41'25.7"N	11°33'45.6"E	543	1.41	0.107	0.27

§ Jafarova, M., Zinicovscaia, I., Yushin, N. Bonini, I., Winkler, A. and Loppi, S. 2025. Biomonitoring of atmospheric deposition of potentially toxic elements at rural sites in Tuscany (Central Italy). Arch Environ Contam Toxicol, 88(1), 29–38.

Table S3. Association (Spearman's rank correlation) between air concentrations (SURF) and deposition of nitrogen (N) across the 33 study sites in Tuscany, central Italy. Boxes indicate significant ($p < 0.05$) correlation. Variables: (1) WD PREC, (2) WD OXN, (3) WD RDN, (4) DD OXN, (5) DD RDN, (6) SURF N-OXN, (7) SURF N_NO×, (8) SURF N-RDN, (9) SURF NO, (10) SURF HNO_3, (11) SURF NH_3, (12) SURF NH4 F, (13) SURF NO_3 C, (14) SURF NO_3 F, (15) SURF NO_2, (16) WD N, (17) DD N, (18) TD OXN, (19) TD N, and (20) N content (%). See Figure S2 for abbreviations.

	2	3	4	5	6	7	8	9	10	11	12	13	14	15	16	17	18	19	20
1	0.000	0.000	0.741	0.001	0.390	0.379	0.038	0.241	0.235	0.016	0.577	0.344	0.561	0.217	0.000	0.029	0.002	0.000	0.695
2		0.001	0.001	0.026	0.006	0.006	0.214	0.005	0.000	0.117	0.823	0.287	0.851	0.003	0.000	0.787	0.000	0.000	0.041
3			0.972	0.067	0.071	0.083	0.584	0.039	0.927	0.321	0.392	0.007	0.017	0.034	0.000	0.301	0.040	0.000	0.689
4				0.826	0.000	0.000	0.962	0.000	0.000	0.632	0.004	0.000	0.171	0.000	0.106	0.000	0.000	0.007	0.002
5					0.787	0.872	0.000	0.783	0.097	0.000	0.014	0.987	0.152	0.692	0.056	0.000	0.056	0.870	0.596
6						0.000	0.516	0.000	0.000	0.935	0.000	0.416	0.000	0.000	0.013	0.002	0.000	0.000	0.092
7							0.403	0.000	0.000	0.892	0.000	0.635	0.000	0.000	0.016	0.004	0.000	0.000	0.130
8								0.483	0.209	0.000	0.001	0.468	0.008	0.518	0.491	0.000	0.185	0.243	0.932
9									0.000	0.999	0.000	0.817	0.000	0.000	0.007	0.005	0.000	0.000	0.173
10										0.108	0.309	0.000	0.861	0.000	0.034	0.038	0.000	0.023	0.005
11											0.040	0.385	0.148	0.953	0.236	0.001	0.092	0.529	0.894
12												0.629	0.000	0.000	0.357	0.000	0.479	0.022	0.606
13													0.105	0.880	0.381	0.048	0.022	0.640	0.002
14														0.000	0.081	0.031	0.913	0.012	0.550
15															0.005	0.009	0.000	0.000	0.141
16																0.709	0.000	0.000	0.347
17																	0.108	0.036	0.047
18																		0.000	0.004
19																			0.139
20																			
